# Phylogenomics illuminates the evolution of bobtail and bottletail squid (order Sepiolida)

**DOI:** 10.1038/s42003-021-02348-y

**Published:** 2021-06-29

**Authors:** Gustavo Sanchez, Fernando Á. Fernández-Álvarez, Morag Taite, Chikatoshi Sugimoto, Jeffrey Jolly, Oleg Simakov, Ferdinand Marlétaz, Louise Allcock, Daniel S. Rokhsar

**Affiliations:** 1 Graduate School of Integrated Science for Life, Hiroshima University, Higashi Hiroshima, Hiroshima Japan; 2 Molecular Genetics Unit, Okinawa Institute of Science and Technology Graduate University, Onna, Okinawa, Japan; 3 Ryan Institute and School of Natural Sciences, National University of Ireland Galway, Galway, Ireland UK; 4 Department of Molecular Evolution and Development, University of Vienna, Vienna, Austria; 5 Department of Genetics, Evolution and Environment, Centre for Life’s Origins and Evolution, University College London, London, UK; 6 Department of Molecular and Cell Biology, Life Sciences Addition #3200, Berkeley, CA USA; 7 Chan-Zuckerberg BioHub, San Francisco, CA USA

**Keywords:** Molecular evolution, Biogeography

## Abstract

Bobtail and bottletail squid are small cephalopods with striking anti-predatory defensive mechanisms, bioluminescence, and complex morphology; that inhabit nektobenthic and pelagic environments around the world’s oceans. Yet, the evolution and diversification of these animals remain unclear. Here, we used shallow genome sequencing of thirty-two bobtail and bottletail squids to estimate their evolutionary relationships and divergence time. Our phylogenetic analyses show that each of Sepiadariidae, Sepiolidae, and the three subfamilies of the Sepiolidae are monophyletic. We found that the ancestor of the Sepiolinae very likely possessed a bilobed light organ with bacteriogenic luminescence. Sepiolinae forms a sister group to Rossinae and Heteroteuthinae, and split into Indo-Pacific and Atlantic-Mediterranean lineages. The origin of these lineages coincides with the end of the Tethys Sea and the separation of these regions during the Eocene and the beginning of the Oligocene. We demonstrated that sepiolids radiated after the Late Cretaceous and that major biogeographic events might have shaped their distribution and speciation.

## Abstract (web summary)

Sanchez, Rokhsar, and colleagues use genome skimming to generate DNA markers for phylogenomic analyses of bobtail and bottletail squid. The ancestral state of bioluminescence suggests that the Sepiolinae ancestor possessed a bilobed light organ with bacteriogenic luminescence. Also, analyses of divergence time suggest that major biogeographic events might have shaped the speciation of these animals.

## Introduction

Sepiolids are small round-bodied cephalopods of around 1–8 cm of dorsal mantle length which live in a range of habitats from shallow coastal waters to mesopelagic environments across the globe^1^. These animals are nektobenthic and pelagic species that have evolved remarkable anti-predatory defense mechanisms. Bobtail squids, for example, use counter-illumination^2^ or discharge luminous secretions^3^, while bottletail squids secrete toxic substances through their slime^4^. Taxonomically, sepiolids comprise the order Sepiolida Keferstein, 1866 which is split into the families Sepiolidae Leach, 1817 and Sepiadariidae Fisher, 1882, commonly known as bobtail and bottletail squid, respectively.

Bobtail and bottletail squids are emerging as model organisms for cephalopods due to their easy culture under laboratory conditions^5^. Most notably, the Hawaiian bobtail squid *Euprymna scolopes* Berry, 1913 is a model for host-symbiont interaction with the bacterium *Allivibrio fischeri* (Beijerinck 1889)^6^, which interacts with the light organ of the animal^2,7,8^. The luminous organs of bobtail squids emit light of bacteriogenic or autogenic origin; these organs have either bilobed or rounded forms^3,9^. Genomic resources to study these animals have also increased through the recent sequencing of the nuclear genome of *E. scolopes*^10^ and transcriptomes of several different species^11–14^.

Previous phylogenetic analyses have placed the order Sepiolida as sister to the family Idiosepiidae Appellöf, 1898^15,16^. The recent study of Anderson and Lindgren 2020^17^, however, finds Sepiolida to be sister to a clade containing the Oegopsida Orbigny, 1845 (oceanic squid with no cornea), Myopsida Naef, 1916 (neritic squid with cornea), and Sepiida Zittel, 1895 (cuttlefish) adding a controversy about the phylogenetic position of these animals. Sepiolida forms the third largest group of Decapodiformes after cuttlefishes and oegopsids^18^. Bobtail squids are the most diverse of the order with around 68 valid species grouped within three subfamilies, Sepiolinae Leach, 1817, Rossinae Appellöf, 1898, and Heteroteuthinae Appellöf, 1898. In contrast, their relatives, the bottletail squids have only five species grouped into two genera, *Sepiadarium* Steenstrup, 1881 and *Sepioloidea* d’Orbigny 1845. The genus *Euprymna* Steenstrup, 1887 has been recently redescribed using both molecular and morphological analyses^11^. The systematics of Sepiolinae has recently been reviewed by Bello^19^, who proposed subdividing *Euprymna* and other genera into multiple new genera according to variation in the shape of the hectocotylus and other morphological characters. However, more molecular studies with confident taxonomic identifications are required to clarify the systematics, diversity, and evolutionary relationships of Sepiolida.

Recently, transcriptome sequencing provided several nuclear DNA markers and resolved the evolutionary relationships of *Euprymna* and other species formerly classified in the genus *Sepiola* Leach, 1817 (see Bello^19^) collected in the shallow waters (up to 20 m depth) of the Ryukyu archipelago and mainland Japan^11^. Other members of the order Sepiolida, however, are found in oceanic waters (the record is for *Heteroteuthis nordopacifica* Kubodera and Okutani, 2011 collected at 1000 m depth, off Toba district in Japan^20^), and are often caught as by-catch in which the animal does not survive the collection. While this makes the sampling of fresh tissue of these animals for transcriptome sequencing difficult, genomic DNA suitable for sequencing is readily recovered.

While full genome sequencing is costly, low-coverage shotgun sequencing allows the recovery of phylogenetically useful markers, including complete or nearly complete mitochondrial genomes and highly copied nuclear loci such as 18S and 28S rRNA^21^. This approach, sometimes called “genome skimming”, does not require the collection of fresh tissues from the animal. For sepiolids, the genome of *E. scolopes*^10^ provides a suitable reference to map reads and recover additional nuclear loci.

In this study, we reconstruct the evolutionary relationships of Sepiolida and performed molecular dating to estimate their divergence timeframe. We first characterized our specimens based on morphological characters, and then sequenced their DNA at low coverage to skim for mitochondrial genes and nuclear ribosomal genes, and to define a set of ultraconserved loci based on shared alignments to the reference genome of *E. scolopes*. Our datasets allowed us to reconstruct mitochondrial and nuclear phylogenetic trees that support a sister relationship between Rossinae and Heteroteuthinae, and the split of Sepiolinae into lineages from the Indo-Pacific and Atlantic plus Mediterranean region. We also infer that the ancestor of Sepiolinae possessed bilobed light organ hosting symbiotic bacteria, lost independently in *Inioteuthis* and *Sepietta* from Sepiolinae. Moreover, the monophyly of Heteroteuthinae confirms a unique origin of the autogenic luminescence in this group, validating its association with pelagic lifestyles in Cephalopoda. In addition, the time-calibrated phylogeny shows that the split of many sepiolids is correlated with and was plausibly driven by major biogeographic events after the late Cretaceous.

## Results and discussion

### Genome skimming provides robust phylogeny

Pioneering molecular phylogenetic studies in Sepiolida that used short regions of a few mitochondrial and nuclear genes failed to resolve the relationship of major clades^9,22,23^. To increase the number of phylogenetically informative sites, Sanchez et al.^11^ sequenced and analyzed the transcriptomes of multiple species of *Euprymna* Steenstrup, 1887, related bobtail squids including *Sepiola parva* Sasaki, 1913 and *Sepiola birostrata* Sasaki, 1918, and several bottletail squids. They found that *S. parva* grouped with the *Euprymna* species to the exclusion of *S. birostrata*, and further morphological analysis led to the formal redefinition of the genus *Euprymna* and the reassignment of *S. parva* Sasaki, 1913 to *Euprymna parva*^11^. The following year, in an exhaustive study of hectocotylus structure, Bello^19^ proposed that *Euprymna* be split back into the original *Euprymna* Steenstrup, 1998 and a newly defined genus, *Eumandya* Bello 2020 that contains *E. parva* Sasaki, 1913 and *E. pardalota* Reid 2011, two taxa whose arms have two rows of suckers rather than four as in other *Euprymna* species. Similarly, Bello introduced a new genus, *Lusepiola* Bello, 2020 that has the effect of renaming *Sepiola birostrata* Sasaki, 1918 as *Lusepiola birostrata*. For clarity, we adopt the finer-grained nomenclature of Bello below, but happily note that *E. parva* and *E. pardalota* have the same abbreviations in both the notation of Sanchez et al.^11^ and Bello^19^.

Sanchez et al.^11^ also emphasized the need for more taxon sampling, careful species assignment, and the inclusion of more informative sites when studying this group of cephalopods. However, the distribution and lifestyle of many lineages of Sepiolida makes the collection of fresh tissue for RNA sequencing very challenging. To overcome this limitation, we sequenced the genomic DNA of several Sepiolida species at shallow coverage up to 3.6× and accessed by this way several mitochondrial and nuclear loci. Most of our samples were carefully identified at the species level based on morphological characters.

We recovered the mitochondrial genomes of the species targeted in this study and annotated the 13 protein-coding genes, 22 tRNAs, and two rRNAs (although only the conserved region of the large and small rRNA was obtained for *Rondeletiola minor* Naef, 1912).

Additionally, we also downloaded the complete mitochondrial genomes of *S. austrinum* and *Idiosepius* sp., and the transcriptome of *E. tasmanica* available in the NCBI database. The transcriptome of *E. tasmanica* was used to extract its complete set of mitochondrial protein-coding genes. We could reconstruct the mitochondrial gene order for all species with complete mtDNA genomes, but we observed no re-arrangement for members of Sepiolidae, and only *Sepiadarium austrinum* deviated from the arrangement seen in all other Sepiadariidae (Fig. S1).

To complement the mitochondrial-based evolutionary history, we also annotated several nuclear loci. As ribosomal gene clusters are present in numerous copies, they were successfully retrieved for almost all the species, except for 28S of the Sepiadariidae sp. specimen, which appeared problematic and was excluded.

By mapping reads to the reference genome of *E. scolopes*, we obtained 3,279,410 loci shared between at least two species and further selected 5215 loci presented in most of our Sepiolidae species, but allowed some missing data in the *Euprymna* + *Eumandya* clade. This was done because the phylogenetic relationships of the *Euprymna* + *Eumandya* species were previously described in detail in Sanchez et al.^11^ using transcriptome data. Out of the 5215 loci, 5164 loci had a per-site coverage ranging between two and five. After trimming and removal of regions without informative sites, 577 loci remained. These ultraconserved loci had lengths ranging between 10 and 690 base pairs (bp), with an average of 65 bp. Our alignment matrix had a length of 37,512 bp and consisted of 16,495 distinct site patterns, and variable sites between 1 and 130 bp with an average value of 7 bp. We expected a low value of variable sites because these regions are highly conserved.

**Fig. 1:**
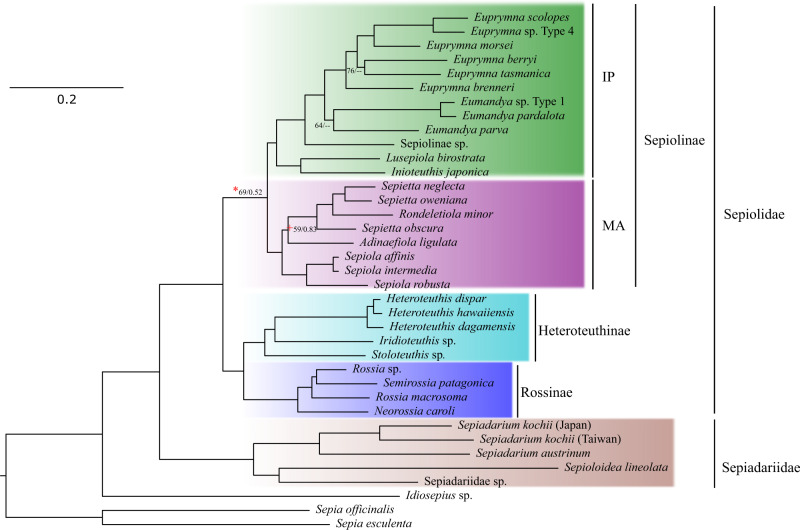
Phylogeny of Sepiolida based on nucleotide sequences from the mitochondria (mt_nc matrix). The topology of the maximum likelihood tree is shown. Numbers by the nodes indicate bootstrap support and the Bayesian posterior probabilities. Values of bootstrap support and posterior probabilities above 95% and 0.95, respectively, are not shown. (*) indicates that the node was resolved with the mito_aa and UCEbob matrices. (+) indicate that *A. ligulata* is sister to *Sepiola* using mito_aa with ultrafast bootstrap support of 94% and a posterior probability of 1. Abbreviations: IP, Indo-Pacific Ocean; MA, Mediterranean Sea, and the Atlantic Ocean.

**Fig. 2:**
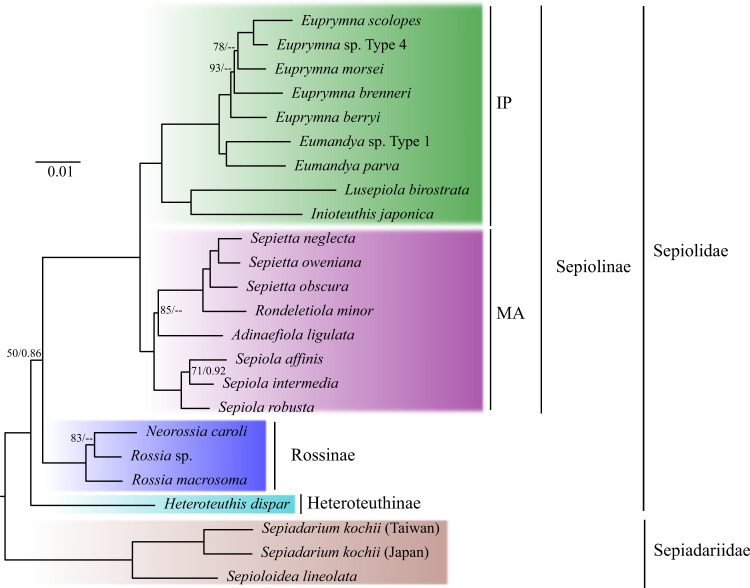
Phylogenetic tree of Sepiolida based on conserved nuclear loci (UCEbob matrix). The topology of the maximum likelihood tree is shown. Numbers in by the nodes indicate the bootstrap support and the Bayesian posterior probability. Values of bootstrap support and posterior probabilities above 95% and 0.95, respectively, are not shown. IP, Indo-Pacific Ocean; MA, Mediterranean Sea, and the Atlantic Ocean.

We considered resolved nodes to be those with the ultrafast bootstrap support and posterior probability larger than 95% and 0.9, respectively. Only the very unresolved nodes were found based on the mito_nc matrix (Fig. 1). However, among the species in these nodes, *Adinaefiola ligulata* Naef, 1912 was well supported with amino acid sequences from mitochondrial genes (posterior probability of 1 and 94% bootstrap support) and partially by the ultraconserved loci (posterior probability of 1, but only 85% bootstrap support) as sister to the *Sepiola* clade (Figs. 2 and S2). Moreover, compared to the mito_nc matrix and with identical topology, mito_aa and UCEbob fully resolved the relationship of the Indo-Pacific and Mediterranean Sea Sepiolinae. The tree generated by the nuclear_rRNA produces a topology with most nodes unsupported (Fig. S3), suggesting these markers are too conserved for assessing the relationships among this group.

Using the UCEbob matrix, the topology and supported relationships of *Euprymna* + *Eumandya* species resemble those reported in Sanchez et al.^11^ using transcriptome sequences, proving our protocol valid when using low coverage sequencing and when a reference genome of the closest related species is available.

The position of *R. minor* showed discordance between mitochondrial and nuclear datasets. Using the mitochondrial matrices, *R. minor* rendered the *Sepietta* Naef, 1912 clade paraphyletic, whereas using the UCEbob and rRNA_nc matrices, *R. minor* appeared sister to the *Sepietta* clade. These relationships were resolved in both mitochondrial and nuclear-based trees and require further investigation with more DNA markers and a wider population sampling.

### Molecular systematics of Sepiolida clades

Using the complete mitochondrial genome, ribosomal nuclear genes, and ultraconserved loci, we recovered the monophyly of the two families of the order Sepiolida—Sepiadariidae and Sepiolidae^9,24^—and the monophyly of the three described subfamilies of the family Sepiolinae. However, contrary to what is proposed based on morphology in Young^24^, the Rossinae is not sister to all the remaining Sepiolidae but rather is sister to Heteroteuthinae, although this is unresolved in the UCE phylogeny. With the lack of systematic work on these subfamilies, our robust phylogenetic backbone in Sepiolida using new samples carefully identified by morphology and with museum vouchers, represents a notable advance to clarify the evolution of morphological traits in major clades within the family.

Based on morphological characters of the hectocotylus, Bello^19^ recently split the polyphyletic Sepiola Leach 1817 into *Lusepiola*, *Adinaefiola*, and *Boletzkyola*, reserving *Sepiola* for the *S. atlantica* group *sensu* Naef 1923. These newly defined clades are consistent with our molecular phylogeny here and in Sanchez^11^, who also noted the polyphyly of *Sepiola* in the Indo-Pacific lineage.

We find that Sepiolinae can be robustly split into two geographically distinct tribes: one that comprises species with known distribution in the Indo-Pacific region (tribe Euprymmini *new tribe*, defined as Sepiolinae with a closed bursa copulatrix, type genus *Euprymna*) and the other including all the Mediterranean and Atlantic species (tribe Sepiolini Appellof, 1989, defined here as Sepiolinae with an open bursa copulatrix, type genus *Sepiola*). Our molecular relationship is consistent with 13 of the 15 apomorphies used in the cladogram shown in Fig. 21 in Bello^19^. The other two proposed apomorphies in Bello (his apomorphic characters 4 and 6) group two IP lineages, *Lusepiola* and *Inioteuthis*, in a clade with species from the Mediterranean and Atlantic. Such relationships contradict our Euprymmini-Sepiolini sister relationship. Moreover, according to our phylogeny, apomorphy 6 of Bello, characterized by the participation of ventral and dorsal pedicels in the formation of the hectocotylus copulatory apparatus, implies that the male ancestor of Sepiolinae had a more developed hectocotylus that was simplified in the *Euprymna* and *Eumandya* clades.

Among euprymins, we confirmed the monophyly of *Euprymna* Steenstrup 1887 as found previously by transcriptome analysis^11^. We also support the monophyly of *Eumandya* Bello, 2020 (Figs. 1 and 2), grouping the type species *E. pardalota* with *E. parva* along with the unnamed “Type 1” Ryukyuan species of Sanchez et al.^11^, for which only hatchlings were available. The phylogenomic grouping of Ryukyuan “Type 1” with *Eumandya* suggests that when its adults are found (or hatchlings are raised to maturity), its arms will carry two rows of suckers. We also found an adult of a Ryukyuan “Type 4” (extending the notation of Sanchez et al.^11^ in the coastal waters of Kume Island, that groups with *E. scolopes* from Hawaii, suggesting a divergence based on geographic isolation in the North Pacific. We also find that *Lusepiola birostrata* (formerly *Sepiola birostrata*) is grouped with *Inioteuthis japonica* as sister to a clade containing *Euprymna*, *Eumandya*, and an unnamed sepioline from Port Kembla, at the northeast of Martin Island in Australian waters.

Among the sepiolins, we confirm the monophyly of *Sepietta* (only for nuclear-genome-based trees, see below). *Adinaefiola*, another genus erected by Bello^19^, with *Sepiola ligulata* Naef 1912 as its type species; was found sister to the *Sepiola* clade, but only in the tree based on amino acid mitochondrial sequences (mito_aa matrix) with a bootstrap value of 94% and a posterior probability of 1 (Fig. S2).

Outside the sepiolines, members of the subfamily Heteroteuthinae are the most elusive and underrepresented in studies of cephalopod systematics due to their oceanic lifestyles. The placement of several heteroteuthin remains controversial. Lindgren et al.^9^, with six nuclear and four mitochondrial genes downloaded from GenBank found that *Sepiolina* Naef, 1912 was sister to *Heteroteuthis* Gray, 1849 + *Rossia* Owen, 1834+ *Stoloteuthis* Verril, 1881; rendering the subfamily Heteroteuthinae polyphyletic. In contrast, our work supports the monophyly of Heteroteuthinae by including *Stoloteuthis* and *Heteroteuthis* in this subfamily, while *Rossia* was placed within the Rossinae (Figs. 1, 2, S2). Members of Heteroteuthinae included in this study formed a sister group to a monophyletic Rossinae (Figs. 1, 2, S2). *Semirossia*, however, rendered the *Rossia* clade paraphyletic. Further discussion about the position of *Semirossia* is difficult because of the lack of information about the original source of this specimen in Kawashima et al.^25^.

### The light organ and luminescence evolution

Bobtail squids are thought to use the bioluminescence of their light organ to camouflage them from predators while foraging and swimming at night through a mechanism called counter-illumination. This has been researched extensively using *E. scolopes* as a model system^26–28^. Unfortunately, the limited number of sequences available and the misidentification of bobtail squids in the GenBank database^11,29,30^ have hindered our understanding of the light organ evolution in the whole taxon.

**Fig. 3:**
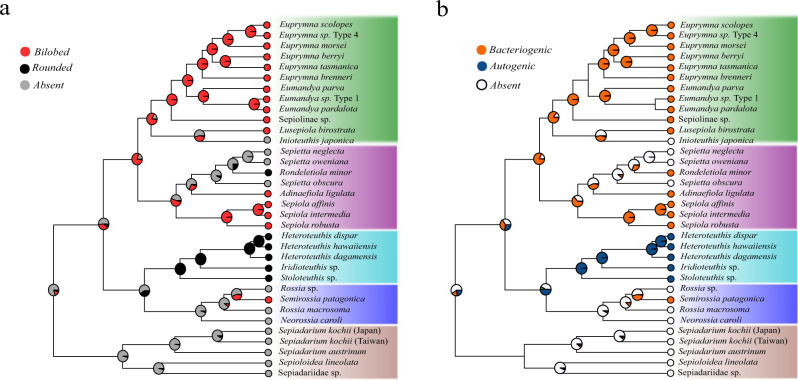
Ancestral character reconstruction (ASR). ASR of (**a**) the shape of the light organ and (**b**) the origin of luminescence in the Sepiolida. The posterior probability of each state is shown as a pie chart, mapped tree generated in BEAST (based on mito_nt matrix, see below), with the outgroups removed.

Our robust phylogeny and Bayesian reconstruction of ancestral bioluminescence clarify how the light organ and its luminescence have evolved in the family Sepiolidae. Members of Sepiolinae comprise neritic and benthic adults with bilobed light organs, except for two genera: *Inioteuthis* from the Indo-Pacific region, and the *Sepietta* species from the Mediterranean Sea and the Atlantic waters. The ancestor of the Sepiolinae very likely possessed a bilobed light organ that harbored luminescent symbiotic bacteria (Fig. 3). This character persisted until the ancestor of the euprymnins and sepiolins. Assuming that *R. minor* is sister to the *Sepietta* clade (as shown with the nuclear-based dataset, Fig. 2), it is clear that the bilobed light organ was lost once in *Inioteuthis* and *Sepietta*, and simplified to a rounded organ in *R. minor*. The alternative scenario, where *R. minor* renders the *Sepietta* clade paraphyletic (based on mitochondrial matrices, Fig. 1), is less plausible as it implies that the light organ was lost twice in the *Sepietta* group, once in *S. obscura* and then in the ancestor of *S. neglecta* and *S. oweniana*; or alternatively that it was lost in the ancestor of *Sepietta*-*Rondelentiola* followed by a reversion of this character in the lineage of *Rondelentiola*.

The light organ is also present in all members of Heteroteuthinae. These bobtails are pelagic as adults, and their light organ appears as a single visceral organ rather than the bilobed form found in nektobenthic Sepiolinae. In contrast to the bacteriogenic luminescence of the light organ in *E. scolopes*^31^, previous studies in *H. dispar*^3^ failed to detect symbiotic bacteria and suggested that the luminescence has an autogenic origin. Thus, it seems plausible that the monophyly of Heteroteuthinae found in our study supports the findings in Lindgren et al.^9^ for convergent evolution of autogenic light organs associated with pelagic lifestyle in many squid, octopus, and *Vampyroteuthis* Chun, 1903^9,32^.

### Divergence time of Sepiolida

**Fig. 4:**
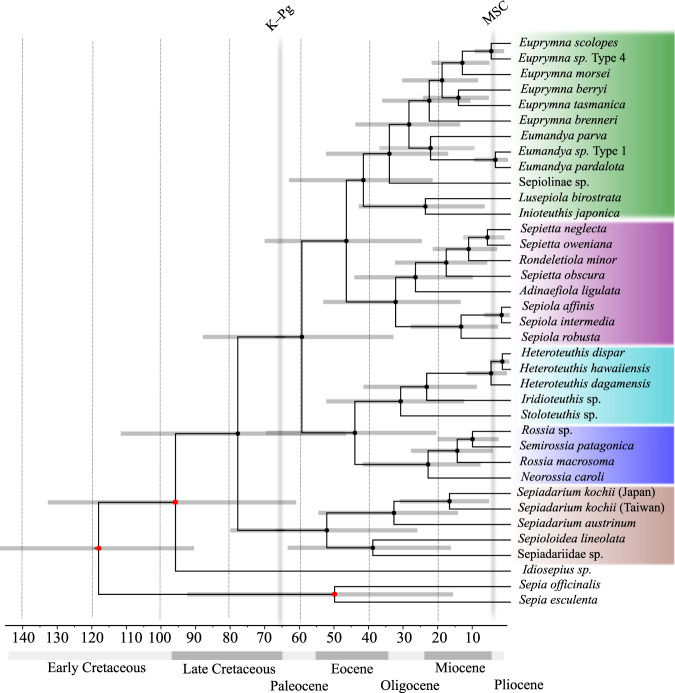
A chronogram of sepiolids using complete mitochondrial genes. Red dots indicate the nodes with secondary calibrations. K-Pg, refers to the Cretaceous-Paleogene boundary and MSC, to the Messinian salinity crisis.

The absence of fossils for this group limited our calculations of divergence time to the use of secondary calibrations. These calibrations can provide more accurate estimates depending on the type of primary calibrations that are used^33^. We retrieved secondary calibrations from previous estimations in Tanner et al.^15^, who used eleven fossil records spanning from coleoids to gastropods in transcriptome-based phylogenetic trees. Specifically, we used the time for the splits of *Sepia esculenta* and *S. officinalis* (~91 Mya), Idiosepiidae, and Sepiolida (~132 Mya) and the origin of the Decapodiformes (root age, ~174 Mya) (Fig. 4). These calibrations and our robust phylogenetic trees allow us to investigate the events that shape the divergence of some clades of the order Sepiolida (Figs. 4,  S4).

Sepiolida appeared before the Cretaceous-Paleogene extinction event^34^, during the middle Mesozoic around 94 Mya (95% HPD = 60.61–130.72). This time frame coincides with the rapid diversification of several oegopsida lineages^15,35^. Our molecular estimates also indicate that radiation of Sepiolidae and Sepidariidae occurred around the Cretaceous-Paleogene boundaries and is concurrent with the rapid diversification of modern marine percomorph fishes around the globe, after the extinction of Mesozoic fishes^36,37^.

Among the species of Sepiolinae collected in the Mediterranean Sea for this study, only *Sepiola robusta* Naef, 1912, and *Sepiola affinis* Naef, 1912 are endemic to the Mediterranean Sea^38^. The distribution of the other species includes the Mediterranean Sea, North Atlantic Ocean, East Atlantic Ocean, and/or up to the Gulf of Cadiz. The confidence intervals for the split between the Mediterranean-Atlantic and Indo-Pacific lineages, and their diversification, overlap during the early Eocene to the beginning of the Oligocene (Figs. 4 and  S4). This time interval coincides with the end of the Tethys Sea, which separated the Indo-Pacific from the Mediterranean and Atlantic region through the Indian-Mediterranean Seaway^39,40^. This separation also influenced the divergence of loliginid clades, coinciding with the split between the Eastern Atlantic plus Mediterranean clade (*Loligo*, *Afrololigo*, *Alloteuthis*) and Indo-Pacific clade (*Uroteuthis* and *Loliolus*) (~55 Mya based on Fig. 2 in Anderson and Marian^41^).

Our chronogram indicates that the ancestor of Sepiolinae arose prior to the early Eocene around 46 Mya (95% HPD = 25.16–69.49) (Fig. 4), already possessing a bilobed light organ hosting luminescent bacteria (Fig. 3). We estimate that the split between *S. affinis* and *S. intermedia* occurred around 2.62 Mya (95% HPD = 0.3–7.4) (Fig. 4) during the end of the Zanclean period, when the Atlantic Ocean refilled the Mediterranean after the Messinian salinity crisis^42,43^. While *S. affinis* is a coastal species with a narrow depth limit, *S. intermedia* inhabits a wider range of deeper waters. It is possible that two populations of their ancestor, each adapted to a different ecological niche and diverged sympatrically in Mediterranean waters, and, after the speciation, *S. intermedia* extended its distribution outside the Mediterranean to the Gulf of Cadiz^44^.

We also estimate that the split between *H. dispar* Rüppell, 1844 and *H. hawaiiensis* (Berry, 1909) occurred around 2.4 Mya (95% HPD = 0.46–5.88), coinciding with the closure of the Isthmus of Panama around 2.8 Mya^45^. Surveys of these species found *H. hawaiiensis* in the North Pacific and *H. dispar* in the North Atlantic Ocean and Mediterranean Sea^46^. A recent speciation event might be the reason for the lack of morphological differences between the two species^46^. Thus, these species may be rendered as cryptic species, a phenomenon increasingly reported in oceanic cephalopods^47^. The sister species of this cryptic species complex, *H. dagamensis* Robson, 1924, appeared before, around 6 Mya, and is reported with broad distribution in the South Atlantic Ocean off South Africa, the Gulf of Mexico, North Atlantic Ocean between Ireland and Newfoundland in Canada, and the South Pacific Ocean off New Zealand^48–50^.

The origin of the *Heteroteuthis* ancestor of *H. dispar*, *H. hawaiiensis*, and *H. dagamensis* can be placed in the Pacific Ocean. After the formation of the Isthmus of Panama, the northern population of *Heteroteuthis* might have split into *H. hawaiiensis* in North Pacific and *H. dispar* in the Atlantic Ocean (from where it also migrated to the Mediterranean Sea). Meanwhile, the formation of the equatorial currents isolated the southern population of *Heteroteuthis* and gave rise to *H. dagamensis*. Then, *H. dagamensis* extended its distribution from the Southern Pacific to the South Atlantic Ocean, the North Atlantic waters, and the Gulf of Mexico. Analysis of molecular species delimitation, however, suggests that *H. dagamensis* includes cryptic lineages among Atlantic and New Zealand populations^30^.

While the origin of *Heteroteuthis* might also be in the Atlantic Ocean, the higher diversity of heteroteuthins in the Pacific (*H. hawaiiensis*, *H. dagamensis*, *H. ryukyuensis* Kubodera, Okutani and Kosuge, 2009, *H. nordopacifica* Kubodera and Okutani, 2011, and an unknown *H. sp*. KER (only known from molecular studies^49^)) than at the Atlantic (*H. dispar* and *H. dagamensis*), make its origin at the Atlantic less plausible. Moreover, the Atlantic *Heteroteuthis* were found nested within Heteroteuthinae species from the Pacific, supporting Pacific Ocean origin (Figs. 1, 4).

By sequencing the genomic DNA of sepiolids at low coverage, we recovered complete mitochondrial genomes and nuclear ribosomal genes for most of our collections. Furthermore, mapping reads to the reference genome of *E. scolopes* allowed us to retrieve additional nuclear-ultraconserved regions. We demonstrate that these nuclear and mitochondrial loci are useful to reconstruct robust phylogenetic trees, especially when the transcriptomes of specimens are difficult to collect, as for sepiolids inhabiting oceanic environments. Finally, our study integrated genomic DNA sequencing with confident morphological identification, which helped to reconstruct the ancestral character of the light organ and its luminescence in sepiolids, and clarify how major lineages have evolved, establishing the existence of distinct Indo-Pacific and Mediterranean-Atlantic subfamilies of Sepiolinae. Our collections and genomically anchored phylogenies will provide a reliable foundation classification of sepiolids for future studies.

## Materials and methods

### Specimens and data collection

For this study, we sampled 32 different species of bobtail and bottletail squids. Samples were collected in different locations of the Japan Sea, Western North Pacific Ocean, Atlantic Ocean, Mediterranean Sea, Tasman Sea, Great Australian Bight, Banda Sea, and South Pacific Ocean. Specimens comprised members of the family Sepiadariidae and all the valid subfamilies of Sepiolidae (Sepiolinae, Heteroteuthinae, and Rossinae). For each individual, a small piece of tissue from the mantle was stored in 70% ethanol for DNA analyses and the remaining animals were stored in museums. Details of our specimens are found in Table S1.

Individuals collected off the coast of mainland Japan and the Ryukyu Archipelago were previously identified in Sanchez et al.^11^. Mediterranean species were identified based on general morphology and/or the hectocotylus following Bello^51^. We also retrieved the full mitochondrial genome of *Semirossia patagonica* (E. A. Smith, 1881) (AP012226) and *Sepiadarium austrinum* Berry, 1921 (KX657686), mitochondrial genes from the transcriptome of *Euprymna tasmanica* (Pfeffer, 1884) (SRR2984339), and the nuclear reads of *Euprymna scolopes* (PRJNA470951) from the GenBank database. For the outgroups, we retrieved the complete mitochondrial genome of *Sepia esculenta* Hoyle, 1885 (NC_009690), *Sepia officinalis* Linné, 1785 (NC_007895) and *Idiosepius* sp. (KF647895), the complete 18S rRNA gene of *Idiosepius pygmaeus* Steenstrup, 1881 (AY557477.1), and a partial region of the 28S rRNA gene of *Idiosepius pygmaeus* (AY293684.1). While the proximal outgroup of the Sepiolidae remains unclear (with different tree topologies found based on transcriptome sequences in Tanner et al^15^, Lindgren and Anderson^52^, and Anderson and Lindgren^17^), we believe the inclusion of Sepiadariidae should prevent any conflict relationships within Sepiolidae.

### DNA isolation, library preparation, and data filtering

We isolated the genomic DNA of our specimens using the DNeasy Blood and Tissue kit (Qiagen). Genomic libraries were constructed using Illumina TruSeq DNA LT Sample Prep Kit. Libraries were size selected to 300 bp, pooled, and sequenced on an Illumina Hiseq 4000 (2 × 150 bp paired-end run). We generated around 1.6–3.6X coverage per species (assuming the total number of reads, the read length, and a genome size of 5 GB reported for *Euprymna scolopes*^10^ (Table S1).

Illumina reads were demultiplexed, and low-quality reads were filtered using Trim Galore v. 0.4.0 (http://www.bioinformatics.babraham.ac.uk/projects/trim_galore/) and adapter-trimmed with *cutadapt*^53^.

### Genome skimming

Bioinformatic analyses were performed on the Okinawa Institute of Science and Technology (OIST) HPC cluster and the National Institute of Genetics cluster.

### Mitochondrial genes

We assembled the mitochondrial genome *de novo* for all our collections using NOVOplasty 4.2^54^ with the cytochrome oxidase I gene of each species or the closest relative species available on the GenBank as a seed and the complete mitochondrial genome of *Idiosepius* sp. Reid and Strugnell 2018 (KF647895) as a reference. The FASTA files recovered were annotated using the standalone ORFfinder (https://www.ncbi.nlm.nih.gov/orffinder/). We selected protein-coding genes per species based on the best hit of their amino-acid sequences with the amino-acid sequences retrieved from the mitochondrial genome of *Idiosepius* sp. using the blastp in the standalone NCBI BLAST v2.10 (https://ftp.ncbi.nlm.nih.gov/blast/executables/blast+/LATEST/). We used the MITOS Web Server^55^ to annotate ribosomal and transfer genes. The information on the gene order from each mitochondrial genome was obtained from the overall results of the MITOS Web Server and ORFfinder.

Protein coding genes were codon-based aligned using MUSCLE 3.8^56^ implemented in AliView^57^ and the borders were trimmed manually. Ribosomal RNAs were aligned using R-coffee^58^ and poorly aligned regions were deleted through Gblocks v0.91b^59^. Transfer RNAs were concatenated for further analyses.

### Nuclear genes

We assembled the nuclear ribosomal genes 28S and 18S using NOVOplasty. The seed used was a conserved region from multiple 18S and 28S sequences retrieved from GenBank and our alignment. The contigs retrieved were annotated in RNAmmer 1.2 Server^60^. Alignment and trimming were performed following the same methodology as the mitochondrial ribosomal genes.

### Ultraconserved loci

We only considered 24 species sequenced with a depth of coverage higher than 2X. Illumina reads were aligned to the reference genome of *Euprymna scolopes* using the *bwa mem* algorithm from the Burrows-Wheeler Aligner (BWA) v0.7.17^61^ with the default mode. We filtered bam files with reads mapped with a quality of at least 40 using Samtools v1.9^62^ and marked duplicate reads with Sambamba v0.7.1^63^. Using these bam files, we generated a list of regions shared between at least two species using PHYLUCE^64^. This list was used to generate a dataset of loci (UCEbob) that are shared in a minimum of 14 species (58%) to recover enough polymorphic sites for the phylogenetic analyses (see below).

For UCEbob, we first created VCF files for each species per shared loci using *freebayes* v.1.3.2^65^ with the settings: -min-coverage 2, -report-monomorphic, -limit-coverage 5, and -use-best-n-alleles 1. Then, VCF files were used to reconstruct aligned FASTA files of DNA sequences using custom scripts. For each locus, we removed sites with gaps presented in more than 50% of the species using Gblocks v0.91b^59^.

### Phylogenetic analysis

Apart from the UCEbob matrix, we also created different alignment matrices: (1) mito_aa: with amino acid sequences from mitochondrial genes, (2) mito_nc: with nucleotide sequences from all coding and non-coding mitochondrial genes, (3) nuclear_rRNA: with the nuclear ribosomal genes.

For each matrix, we performed maximum likelihood (ML) phylogeny in IQ-TREE^66^ with the best model and partition scheme selected by ModelFinder^67^, and 1000 replicates of ultrafast likelihood bootstrap^68^. Additionally, we performed a phylogenetic inference with a Bayesian approach in Exabayes v.1.5^69^ for all matrices using a partition per gene and the GTR + Gamma model of substitution. Two independent chains of more than 1.5 × 10^6^ generations were run in parallel, sampling every 1000 generations. The chains’ convergence was validated when the average standard deviation from split frequencies was less than 0.5% and the ESS more than 200. Finally, we discarded the first 25% generations as burn-in, and the 50% majority consensus tree was summarized using the Consensus script from Exabayes.

### Ancestral character reconstruction

We used BayesTraits v3^70^ with the multistate model of discrete traits to obtain the posterior probability of the light organ shape and the luminescence origin of ancestral sepiolids. We coded the shape of the light organ as bilobed, rounded, and absent; and luminescence origin as autogenic, bacteriogenic, and absent. For taxa with no information about the state of the trait analyzed, we assume such a state is the same as its congeneric species.

We applied the reversible jump MCMC model with hyper prior exponential distribution of interval from 0 to 30, as this prior produces the best acceptance rate (average of 30%). As the input trees, we used 24335 trees generated from our divergence time analysis in BEAST (see below) after 25% of burn-in. We run BayesTraits with 1 million interactions, sampling every 1000th iteration; and plot the mean probabilities of each character in the ultrametric tree generated with the mito_nt matrix using the R package ape^71^.

### Divergence time

Divergence time for several lineages within Sepiolida was estimated using a Bayesian approach implemented in BEAST v2.6^72^ with nucleotides sequences of the mitochondrial gene (the mito_nc matrix). The analysis in BEAST was run with the best model of substitution for each partition selected by bmodeltest^73^, the Yule model of speciation, and the fast relaxed lognormal clock. We used internal calibrations estimated in Fig. S4 of Tanner et al.^15^ as priors with the normal distribution and the mean in real space as follows: Decapodiformes Leach, 1817 (mean: 174.22 Mya; SD: 26), Sepiida Zittel, 1895 (mean: 91.13 Mya; SD: 20; and “use originate”), and for the split of Idiosepiidae Appellof, 1898 and Sepiolida (mean: 132.37 Mya; SD: 26; and “use originate”). The analysis was run twice for 160 million generations, sampling every 5000 generations. The convergence of ESS > 200 was evaluated in Tracer v.1.7.1^74^ and the chronogram was annotated in TreeAnnotator using the topology of the maximum likelihood tree constructed with mito_nc matrix, with a burn-in of 10% and mean node heights.

### Reporting summary

Further information on research design is available in the Nature Research Reporting Summary linked to this article.

## Supplementary information

Supplementary Information

Reporting Summary

## Data Availability

Input and output for analyses in BayesTraits, and alignments, best model and partition scheme for each matrix can be found in FigShare (https://figshare.com/s/1e0decb1d073a34fee2a). Raw reads can be found in the GenBank database under the BioProject number PRJNA640585.
