# Supplementary material for: Phylogenomics illuminates the evolution of bobtail and bottletail squid (order Sepiolida)
Source: Commun Biol. 2021 Jun 29;4:819. doi: 10.1038/s42003-021-02348-y (PMC8241861; doi:10.1038/s42003-021-02348-y)
Supplement: Supplementary file 1 — Supplementary Information [file 42003_2021_2348_MOESM1_ESM.pdf]

Phylogenomics illuminates the evolution of bobtail and bottletail squid (order Sepiolida)

Gustavo Sanchez [1,2]*, Fernando Á. Fernández-Álvarez [3]*, Morag Taite [3], Chikatoshi Sugimoto [2], Jeffrey Jolly [2], Oleg Simakov [4], Ferdinand Marlétaz [5], Louise Allcock [3], Daniel S. Rokhsar [2,6,7]+

[1] Graduate School of Integrated Science for Life, Hiroshima University, Higashi Hiroshima, Hiroshima, Japan

[2] Molecular Genetics Unit, Okinawa Institute of Science and Technology Graduate University, Onna, Okinawa 904-0495, Japan

[3] Ryan Institute and School of Natural Sciences, National University of Ireland Galway, Galway, Ireland

[4] Department of Molecular Evolution and Development, University of Vienna, Vienna, Austria

[5] Centre for Life's Origins and Evolution, Department of Genetics, Evolution and Environment, University College London, United Kingdom

[6] Department of Molecular and Cell Biology, Life Sciences Addition #3200, Berkeley, California 94720-3200, USA

[7] Chan-Zuckerberg BioHub, 499 Illinois Street, San Francisco CA 94158, USA

*These authors contributed equally
+Corresponding author, e-mail: dsrokhsar@gmail.com

SUPPLEMENTARY INFORMATION

Supplementary Figures:

Supplementary Figure 1. Mitochondrial gene order of members from the Sepiolidae.
Supplementary Figure 2. Phylogenetic relationship of the Sepiolidae using mitochondrial amino-acid sequences.
Supplementary Figure 3. Phylogenetic relationship of the Sepiolidae using concatenated nuclear ribosomal genes.
Supplementary Figure 4. Phylogenetic chronogram of the Sepiolidae.

Supplementary Tables:
Supplementary Table 1. Information about the specimens used in this study and the corresponding museum catalog numbers, and GenBank accession numbers.

Figure S1. Representative mitochondrial gene arrangements of the sepiolids used in this study. Only *S. austrinum* present different gene order within the Sepiadariidae. All specimens from the Sepiolidae share the same gene order. (*) indicates that the mitochondrial contig of *S. austrinum* was retrieved from the Genbank database. (+) indicates the gene order of the Sepiadariidae minus *S. austrinum*.

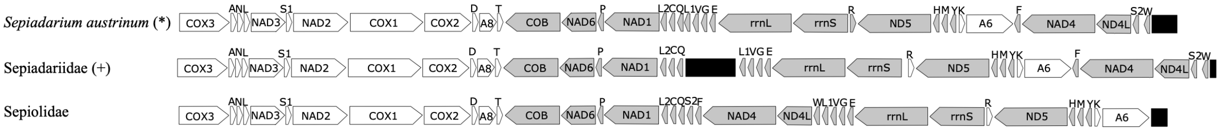

Figure S2. Phylogenetic relationship of the Sepiolidae based on mitochondrial amino-acid sequences (mito_aa matrix). The topology of the maximum likelihood tree is shown. Numbers in the nodes represent the ultrafast bootstrap support and the Bayesian posterior probabilities. Values of bootstrap support and posterior probabilities above 95% and 0.95, respectively, are not shown.

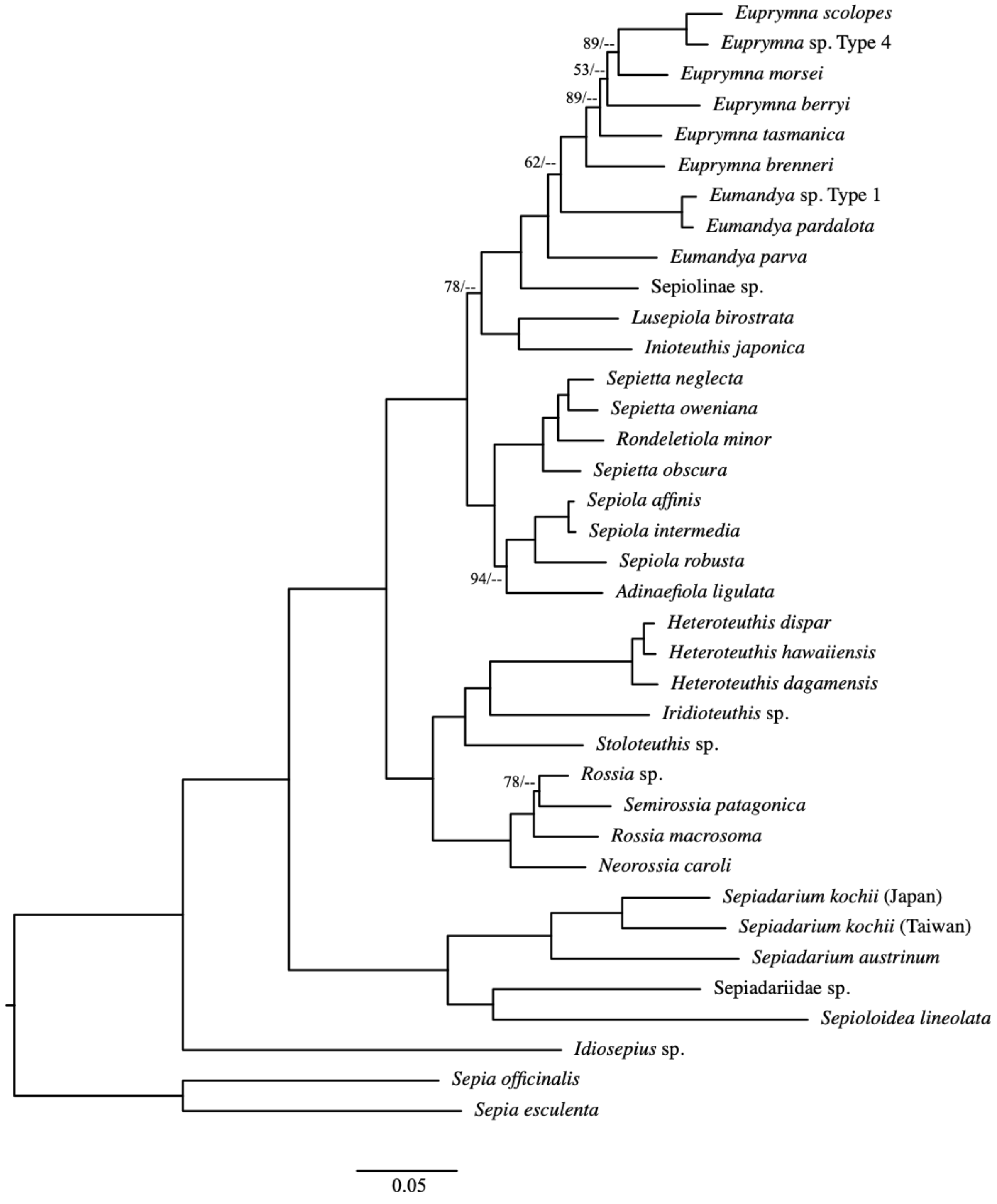

Figure S3. Phylogenetic relationship of the Sepiolidae based on the concatenated nuclear ribosomal genes (nuclear_rRNA matrix). The topology of the maximum likelihood tree is shown. Numbers in the nodes represent the ultrafast bootstrap support and the Bayesian posterior probabilities. Values of bootstrap support and posterior probabilities above 95% and 0.95, respectively, are not shown.

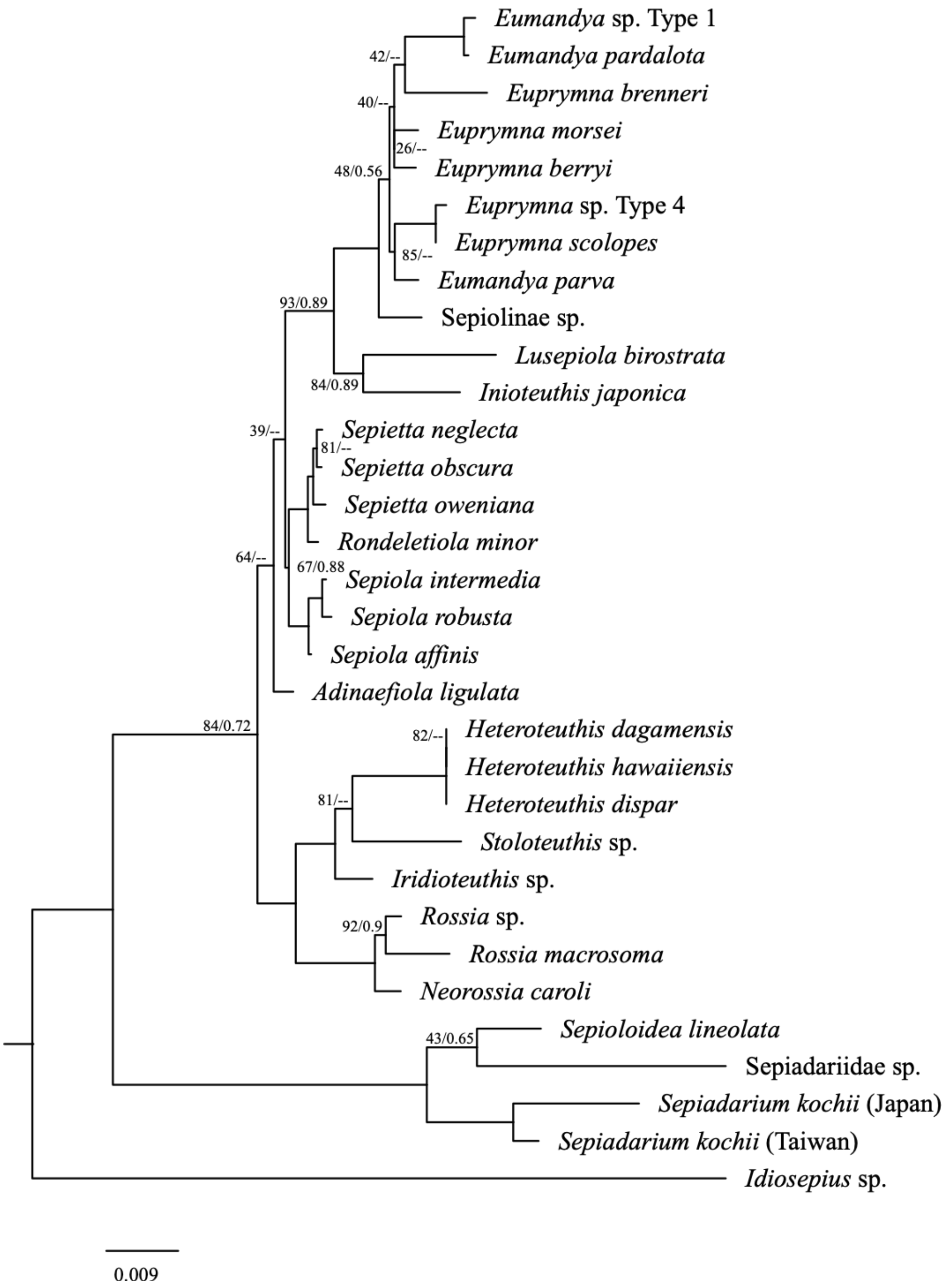

Figure S4. Time-calibrated phylogeny of the Sepiolidae. Values in the nodes represent the 95% credible intervals (95% HPD). A time-scale in a million years is shown at the bottom of the tree.

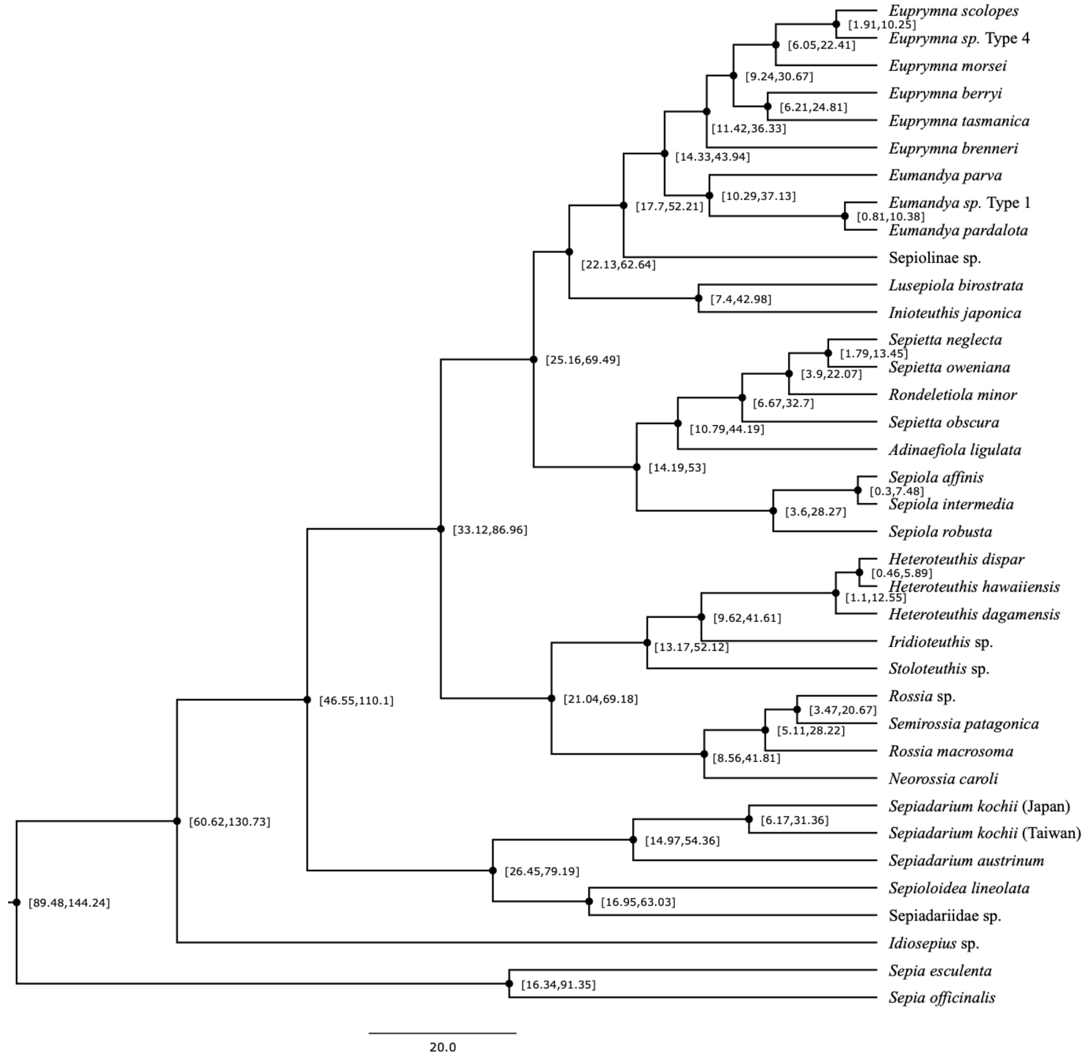

Supplementary Table S1. Information about sepiolid specimens used in this study.

Species	Paired-end reads	Museum Voucher ID	Accession Number Mitochondria	Accession Number nuclear rRNA		Location
				18S	28S	
Euprymna sp. Type 4	600,00,000	AM C.587222	MW478821	LC597609	LC597645	Kumejima, Okinawa, Japan
Euprymna brenneri	56,631,098	NSMT Mo 85891	MW478824	LC597608	LC597637	Miyagi, Okinawa, Japan
Euprymna parva	57,657,362	AM C.575209	MW478826	LC597611	LC597643	The East China Sea, off Zamami Island, Japan
Euprymna pardalota	31,027,818	C.476100.002	MW478833	LC597606	LC597638	East Timor
Sepiolinae sp.	24,355,876	C.553305.001	MW478831	LC597612	LC597642	Port Kembla, northeast of Martin Island, Australia
Inioteuthis japonica	57,812,018	AM C. 587219	MW478829	LC597605	LC597628	The Pacific Ocean, off Niigata Prefecture in mainland Japan
Lusepiola birostrata	56,440,191	AM C.587218	MW478837	LC597615	LC597627	OsakiShimozima, mainland Japan
Sepiola affinis	57,940,641	ICMC000360	MW478836	LC597622	LC597635	The Mediterranean Sea, off Arenys de Mar
Sepiola intermedia	47,278,587	ICMC000363	MW478839	LC597623	LC597636	The Mediterranean Sea, off Tarragona
Adinaefiola ligulata	58,468,596	ICMC000381	MW478840	LC597616	LC597629	The Mediterranean Sea, off Tarragona
Sepietta neglecta	58,113,999	ICMC000356	MW478838	LC597620	LC597631	The Mediterranean Sea, off Tarragona
Sepietta obscura	57,868,835	ICMC00035	MW478841	LC597619	LC597633	The Mediterranean

		5				Sea, off Tarragona
Sepietta oweniana	57,680,214	ICMC00034 0	MW478842	LC597618	LC597630	The Mediterranean Sea, off Tarragona
Sepiola robusta	58,079,658	ICMC00037 4	MW478843	LC597621	LC597634	The Mediterranean Sea, off Tarragona
Rondelentiola minor	56,743,771	ICMC00038 1	MW478846, MW484940	LC597617	LC597632	The Mediterranean Sea, off Tarragona
Heteroteuthis dispar	54,450,019	ICMC00039 3	MW470866	LC597601	LC597648	The Mediterranean Sea, near the Balearic Island
Heteroteuthis dagamensis	24,325,300	NIWA11860 4A	MW478822	LC597600	LC597650	South Pacific Ocean, off Kermadec Island
Heteroteuthis hawaiiensis	22,199,197	C.500820.00 1	MW478828	LC597599	LC597649	Great Australian Bight
Iriodioteuthis sp.	26,314,472	C.500885.00 1	MW478830	LC597598	LC597646	Great Australian Bight
Stoloteuthis sp.	5,590,899	DEO506 (Sta. 3); NMNH	MW478844	LC597597	LC597647	North Atlantic Ocean, Bear Seamount
Neorossia caroli	58,301,416	ICMC00033 0	MW478832	LC597604	LC597624	The Mediterranean Sea, off Vilanova I la Geltru
Rossia macrosoma	57,898,120	ICMC00032 9	MW478834	LC597602	LC597625	The Mediterranean Sea, off Tarragona
Rossia sp.	58,484,581	AM C.587214	MW478835	LC597603	LC597626	The sea of Japan, off Oki island
Sepiadarium kochii (Japan)	57,675,400	AM C.587220	MW484942	LC597596	LC597652	The Pacific Ocean, off Mie Prefecture in mainland Japan

Sepiadarium kochii (Taiwan)	55,196,982	AM C.575210	MW484943	LC597595	LC597653	Penghu Island, Taiwan
Sepiadariidae sp.	35,336,005	NIWA 95127	MW484941	LC597593	-	Between Stewart Island and Aukland Island
Sepioloidea lineolata#	56,836,822	-	MW484944	LC597594	LC597651	Monterey Bay Aquarium
Euprymna berryi#	57,104,355	-	MW478823	LC597613	LC597640	Yamaguchi, Prefecture, mainland Japan
Euprymna morsei#	60,000,000	-	MW478825	LC597614	LC597641	Mie Prefecture, mainland Japan
Euprymna sp. Type 1+	57,254,461	-	MW478827	LC597607	LC597639	Okinawa, Japan
Euprymna scolopes	60,000,000	-	MW478845	LC597610	LC597644	

Abbreviations of museum voucher IDs: NSMT, National Museum of Science and Technology, Tokyo; AM and C, Australian Museum, Sydney, Australia; ICMC, Biological Reference Collections (CBR-ICM) at the Institut de Ciències del Mar of Barcelona (ICM-CSIC); NIWA, The National Institute of Water and Atmospheric Research, New Zealand; and NMNH, Smithsonian National Museum of Natural History.

Symbols indicate:

+, DNA was sequenced from a hatchling.

#, These species were previously identified in Sanchez et al. (2019) and raised in the laboratory of the Molecular Genetics Unit at the Okinawa Institute of Science and Technology. One individual spawned by each of these species was sequenced for this study. We lodged an adult *S. lineolata*, sibling of the specimen sequenced here, at the Australian Museum under voucher id: AM C.574778
