# Supplementary material for: Phylogenomics illuminates the evolution of bobtail and bottletail squid (order Sepiolida)
Source: Commun Biol. 2021 Jun 29;4:819. doi: 10.1038/s42003-021-02348-y (PMC8241861; doi:10.1038/s42003-021-02348-y)
Supplement: Supplementary file 2 — Reporting Summary [file 42003_2021_2348_MOESM2_ESM.pdf]

nature research

Corresponding author(s): Daniel S. Rokhsar

Last updated by author(s): Apr 22, 2021

Reporting Summary

Nature Research wishes to improve the reproducibility of the work that we publish. This form provides structure for consistency and transparency in reporting. For further information on Nature Research policies, see our Editorial Policies and the Editorial Policy Checklist.

Statistics

For all statistical analyses, confirm that the following items are present in the figure legend, table legend, main text, or Methods section.

n/a	Confirmed	
x	☐	The exact sample size (*n*) for each experimental group/condition, given as a discrete number and unit of measurement
x	☐	A statement on whether measurements were taken from distinct samples or whether the same sample was measured repeatedly
x	☐	The statistical test(s) used AND whether they are one- or two-sided<br>*Only common tests should be described solely by name; describe more complex techniques in the Methods section.*
x	☐	A description of all covariates tested
x	☐	A description of any assumptions or corrections, such as tests of normality and adjustment for multiple comparisons
x	☐	A full description of the statistical parameters including central tendency (e.g. means) or other basic estimates (e.g. regression coefficient) AND variation (e.g. standard deviation) or associated estimates of uncertainty (e.g. confidence intervals)
x	☐	For null hypothesis testing, the test statistic (e.g. *F*, *t*, *r*) with confidence intervals, effect sizes, degrees of freedom and *P* value noted<br>*Give P values as exact values whenever suitable.*
☐	x	For Bayesian analysis, information on the choice of priors and Markov chain Monte Carlo settings
x	☐	For hierarchical and complex designs, identification of the appropriate level for tests and full reporting of outcomes
x	☐	Estimates of effect sizes (e.g. Cohen's *d*, Pearson's *r*), indicating how they were calculated

Our web collection on statistics for biologists contains articles on many of the points above.

Software and code

Policy information about availability of computer code

Data collection	Correct species assignments of our specimens were verified by morphological analyses. Code of the museum vouchers of these specimens can be found in Supplementary materials. To generate genetic data, we sequenced these specimens at low coverage.
Data analysis	We use PHYLUCE, freebayes v.1.3.2, IQ-TREE 2.0.3, ModelFinder, and Exabayes v.1.5.

For manuscripts utilizing custom algorithms or software that are central to the research but not yet described in published literature, software must be made available to editors and reviewers. We strongly encourage code deposition in a community repository (e.g. GitHub). See the Nature Research guidelines for submitting code & software for further information.

Data

Policy information about availability of data

All manuscripts must include a data availability statement. This statement should provide the following information, where applicable:

- Accession codes, unique identifiers, or web links for publicly available datasets
- A list of figures that have associated raw data
- A description of any restrictions on data availability

Input and output for analyses in BayesTraits, and alignments, best model and partition scheme for each matrix can be found in FigShare (https://figshare.com/s/1e0decb1d073a34fee2a).
Raw reads can be found in the GenBank database under the BioProject number PRJNA640585.

Field-specific reporting

Please select the one below that is the best fit for your research. If you are not sure, read the appropriate sections before making your selection.

☐ Life sciences ☐ Behavioural & social sciences ☒ Ecological, evolutionary & environmental sciences

For a reference copy of the document with all sections, see nature.com/documents/nr-reporting-summary-flat.pdf

Ecological, evolutionary & environmental sciences study design

All studies must disclose on these points even when the disclosure is negative.

Study description	We assess the evolutionary relationship and divergence time-frame of thirty-two bobtail and bottletail squids using genome skimming.
Research sample	bobtail and bottletail squids of the order Sepiolida.
Sampling strategy	Samples were collected by different fishing methods and water diving.
Data collection	Shotgun sequencing up to 3.6X
Timing and spatial scale	Samples were collected during several research cruise, and by diving from 2017 to 2019.
Data exclusions	No data were excluded.
Reproducibility	The experiment can be repeated using the alignment matrix we provided in FigShare.
Randomization	Samples were identify by morphology and placed in their correct species.
Blinding	Blinding is not relevant for phylogenomic studies.

Did the study involve field work? ☒ Yes ☐ No

Field work, collection and transport

Field conditions	We used collections reported Sanchez et al., 2019 (published also in Communications Biology), and other samples were collected during different research cruises.
Location	Mainland Japan, New Zealand, Atlantic Ocean, Mediterranean Sea.
Access & import/export	n/a
Disturbance	n/a

Reporting for specific materials, systems and methods

We require information from authors about some types of materials, experimental systems and methods used in many studies. Here, indicate whether each material, system or method listed is relevant to your study. If you are not sure if a list item applies to your research, read the appropriate section before selecting a response.

Materials & experimental systems

n/a	Involved in the study	
☒	☐	Antibodies
☒	☐	Eukaryotic cell lines
☒	☐	Palaeontology and archaeology
☐	☒	Animals and other organisms
☒	☐	Human research participants
☒	☐	Clinical data
☒	☐	Dual use research of concern

Methods

n/a	Involved in the study	
☒	☐	ChIP-seq
☒	☐	Flow cytometry
☒	☐	MRI-based neuroimaging

Animals and other organisms

Policy information about studies involving animals; ARRIVE guidelines recommended for reporting animal research

Laboratory animals	the study did not involved laboratory animals.
Wild animals	The specimens used in this study were stored in different museums (see Supplementary Material for details of code and locations).
Field-collected samples	n/a
Ethics oversight	n/a

Note that full information on the approval of the study protocol must also be provided in the manuscript.
